# Serum fibroblast growth factor 19 serves as a potential novel biomarker for hepatocellular carcinoma

**DOI:** 10.1186/s12885-019-6322-9

**Published:** 2019-11-12

**Authors:** Takahiro Maeda, Hiroaki Kanzaki, Tetsuhiro Chiba, Junjie Ao, Kengo Kanayama, Susumu Maruta, Yuko Kusakabe, Tomoko Saito, Kazufumi Kobayashi, Soichiro Kiyono, Masato Nakamura, Sadahisa Ogasawara, Eiichiro Suzuki, Yoshihiko Ooka, Shingo Nakamoto, Ryo Nakagawa, Ryosuke Muroyama, Tatsuo Kanda, Hitoshi Maruyama, Naoya Kato

**Affiliations:** 1 Department of Gastroenterology, Graduate School of Medicine, Chiba University, 1-8-1 Inohana, Chuo-ku, Chiba, 260-8670 Japan; 2 Department of Molecular Virology, Graduate School of Medicine, Chiba University, 1-8-1 Inohana, Chuo-ku, Chiba, 260-8670 Japan; 3 Department of Gastroenterology and Hepatology, Nihon University School of Medicine, 30-1 Oyaguchi-Kamicho, Itabashi-ku, Tokyo, 173-8610 Japan; 4 Department of Gastroenterology, Juntendo University School of Medicine, 2-1-1 Hongo, Bunkyo-ku, Tokyo, 113-8421 Japan

**Keywords:** FGF19, HCC, Tumor marker, AFP, RFA

## Abstract

**Background:**

Abnormal autocrine fibroblast growth factor 19 (FGF19) production has been observed in several types of cancers, including hepatocellular carcinoma (HCC). In this study, we investigated the potential of serum FGF19 as a novel tumor marker of HCC based on a sandwich enzyme-linked immunosorbent assay (ELISA).

**Methods:**

The serum FGF19 levels of 304 patients with HCC was measured by ELISA. The serum levels of existing markers, including alpha-fetoprotein (AFP) and des-gamma-carboxy prothrombin (DCP) were determined by chemiluminescence enzyme immunoassay. Both diagnostic value of FGF19 and its changes after curative ablation therapy was further examined.

**Results:**

The median FGF19 levels in controls, chronic liver disease patients, and primary HCC patients, were 78.8 pg/mL, 100.1 pg/mL, and 214.5 pg/mL, respectively. The subsequent receiver operating characteristic curves (ROC) successfully determined an optimal cut-off value of 200.0 pg/mL. The area under the ROC curve (AUC) of FGF19 for HCC detection was comparable to those of AFP and DCP. Of importance, FGF19 showed higher sensitivity for the detection of small HCC (solitary cancer with diameter < 20 mm) than those of existing markers. In addition, 43 out of 79 cases (54.4%) with normal AFP and DCP (so-called “double negative HCC”) exhibited serum FGF19 level ≥ 200 pg/mL. In 45 HCC patients treated with curative ablation therapy, serum FGF19 levels changed from 257.4 pg/mL to 112.0 pg/mL after the treatment.

**Conclusion:**

Our findings reveal that FGF19 can be a potential novel biomarker for HCC. Although FGF19 is not necessarily a substitute for existing markers, it may help improve the prognosis in HCC patients owing to its resourceful use in various aspects of HCC management and treatment.

## Background

Hepatocellular carcinoma (HCC) is the third largest cause of cancer deaths globally, and the number of new HCC cases has been gradually increasing [1, 2]. In spite of the advances in imaging technology and therapeutic approaches, the 5-year survival rate remains at 20% [3, 4]. Tumor marker detection has been widely used for several purposes, such as comprise diagnosis, follow-up of the post-treatment clinical course, optimization of the treatment efficacy, and prediction of prognosis in a variety of cancers [5]. In HCC, three tumor markers, namely alpha-fetoprotein (AFP), AFP-L3, and des-gamma-carboxy prothrombin (DCP), have been used as serum biomarkers. Although the measurement of these markers is not necessarily needed for the establishment of a definitive diagnosis of HCC as per the guidelines proposed by American Association for the Study of Liver Diseases (AASLD), European Association for the Study of the Liver (EASL), and The Japan Society of Hepatology (JGH), these markers play a key role in monitoring for HCC onset and recurrence [6–8]. However, it is well-known that these markers often remain in the normal range, particularly in small HCC [9]. Furthermore, an unexpected elevation in these markers is sometimes observed in chronic liver disease (CLD) patients who do not have HCC. Taken together, alternative serum biomarkers with high sensitivity and specificity are required.

Fibroblast growth factors (FGFs) signal through FGF receptor (FGFR) tyrosine kinases to regulate a wide range of biological processes, including cell growth, differentiation, angiogenesis, and metabolism [10–12]. It is noteworthy that dysregulated FGF/FGFR signaling contributes to cancer development in many types of cancers [13–18]. Fibroblast growth factor 19 (FGF19), secreted from ileum, negatively regulates bile duct acid synthesis in the liver through FGFR4 activation [19, 20]. However, FGF19 production in an autocrine fashion reportedly activates FGF19/FGFR4 signaling and contributes to HCC development [21]. It has been also demonstrated that the overexpression of FGF19 and FGFR4 is associated with unfavorable prognosis in HCC patients [22]. These findings are mainly based on pathological studies, and whether serum FGF19 functions as a biomarker in HCC remains unclear.

In this study, we conducted a sandwich enzyme-linked immunosorbent assay (ELISA) to examine the serum levels of FGF19 in HCC patients. To determine the sensitivity and specificity of FGF19 as a tumor marker for HCC, the sera of controls and CLD patients were also examined. After determining of the optimal cut-off value based on the receiver operating characteristic curves (ROC), we compared the tumor detection ability of FGF19 and the existing markers. Further, we attempted to determine whether FGF19 functions as a marker of treatment efficacy in HCC treated with ablation therapy.

## Methods

### Patients and blood samples

Blood samples were collected from 304 patients who underwent initial treatment for HCC at the Chiba University hospital between January 2014 and December 2017. Serum samples were collected during the 1-month period before treatment initiation. To investigate the changes in the FGF19 levels, the sera of patients treated with radiofrequency ablation (RFA) were also collected. Similarly, the sera of 142 CLD patients without HCC and those of 10 non-CLD patients (controls) were also collected. After obtaining informed written consent, we analyzed the preserved blood samples and the data acquired from their medical records. This study was approved by the Research Ethics Committees of the Graduate School of Medicine, Chiba University (approval number: 3024).

Primary HCC patients comprised 213 men (70.1%) and 91 women (29.9%), with a median age of 72 years. Liver damage was attributed to Hepatitis B virus (HBV) infection (11.2%), Hepatitis C virus (HCV) infection (51.3%), and others (37.5%) (Table 1). As per the Child–Pugh classification, 80.9% of the patients belonged to class A, 16.1% to class B, and 3.0% to class C. According to the Union for International Cancer Control (UICC) stages, the number of patients in stage I, II, III, and IV were 141 (46.4%), 72 (23.7%), 69 (22.7%), and 22 (7.2%), respectively. The CLD patients comprised 84 men (59.2%) and 58 women (40.8%), with a median age of 65 years. Liver damage was attributed to HBV infection (18.3%), HCV infection (48.6%), and others (33.1%). As per the Child–Pugh classification, 88.0% of the CLD patients belonged to class A, 9.2% to class B, and 2.8% to class C.

**Table 1:** Baseline characteristics of study patients

Characteristics	CLD patients (*n* = 142)	HCC patients (*n* = 304)	*p* value
Age (year)^a^	65 (14)	72 (14)	< 0.001
Sex (male/female)	84/58	213/91	0.023
Etiology (HBV/HCV/Others)	26/69/47	34/156/114	0.116
Liver damage (CH/LC)	66/76	52/252	< 0.001
AFP (ng/mL)^a^	3.8 (3.1)	15.7 (112.7)	< 0.001
DCP (mAU/mL)^a^	20 (9)	93 (1140)	< 0.001
AST (IU/L)^a^	27 (23)	47 (44)	< 0.001
ALT (IU/L)^a^	22 (28)	35 (32)	< 0.001
ALB (g/dL)^a^	4.3 (0.5)	3.7 (0.7)	< 0.001
T-Bil (mg/dL)^a^	0.9 (0.5)	0.9 (0.6)	0.612
PLT (× 10^4^/μL)^a^	14.2 (10.2)	11.9 (9.7)	< 0.001
PT (%)^a^	99 (21)	93 (24)	0.014
Child-Pugh (A/B/C)	125/13/4	246/49/9	0.137

^a^ Data are expressed as median (interquartile range)

Abbreviations, *CLD* chronic liver disease, *HCC* hepatocellular carcinoma, *HBV* hepatitis B virus, *HCV* hepatitis C virus, *CH* chronic hepatitis, *LC* liver cirrhosis, *AFP* alpha-fetoprotein, *DCP* des-gamma-carboxy prothrombin, *AST* aspartate aminotransferase, *ALT* alanine aminotransferase, *ALB* albumin, *T-Bil* total bilirubin, *PLT* platelet, *PT* prothrombin time

### Diagnosis of CLD and HCC

The diagnosis of CLDs, including chronic hepatitis and cirrhosis was based on the laboratory data, clinical manifestation, and/or histological finding [23]. HCC was diagnosed on the basis of contrast-enhanced imaging findings and/or histological analysis as per the diagnostic criteria of the AASLD [24, 25]. A solitary lesion with a diameter < 20 mm defined small HCC.

### Measurement of serum FGF19, AFP, and DCP levels

Serum FGF19 levels of the primary HCC patients, CLD patients, and controls were determined using a sandwich ELISA according to the manufacturer’s instructions (R&D Systems, Inc., MN). Serum AFP and DCP levels were determined by chemiluminescence enzyme immunoassay (CLEIA) (LUMIPULSE® L2400, FUJIREBIO INC., Japan). Serum FGF19 levels of the HCC patients were measured using the sera collected during the 1-month period before the initial treatment. Moreover, the serum FGF19 levels of 45 patients treated with complete ablation were also analyzed at some point in recurrence-free period based on radiological findings.

### Statistical analyses

Data are expressed as the median and interquartile range (IQR) values. Statistical differences in the quantitative valuables of the groups were determined using Wilcoxon rank sum test and Kruskal-Wallis test. Chi-square test was used for the categorical values. Correlation between each marker was determined using Spearman’s rank correlation coefficient. The area under the curve (AUC) values were determined with the ROC analysis. Recurrence-free survival (RFS) was calculated using the Kaplan-Meier method and compared using log-rank test. The level of significance was set at *p* < 0.05. All statistical analyses were performed using the SPSS statistical software package (SPSS version 24).

## Results

### Serum FGF19 levels

**Fig. 1:**
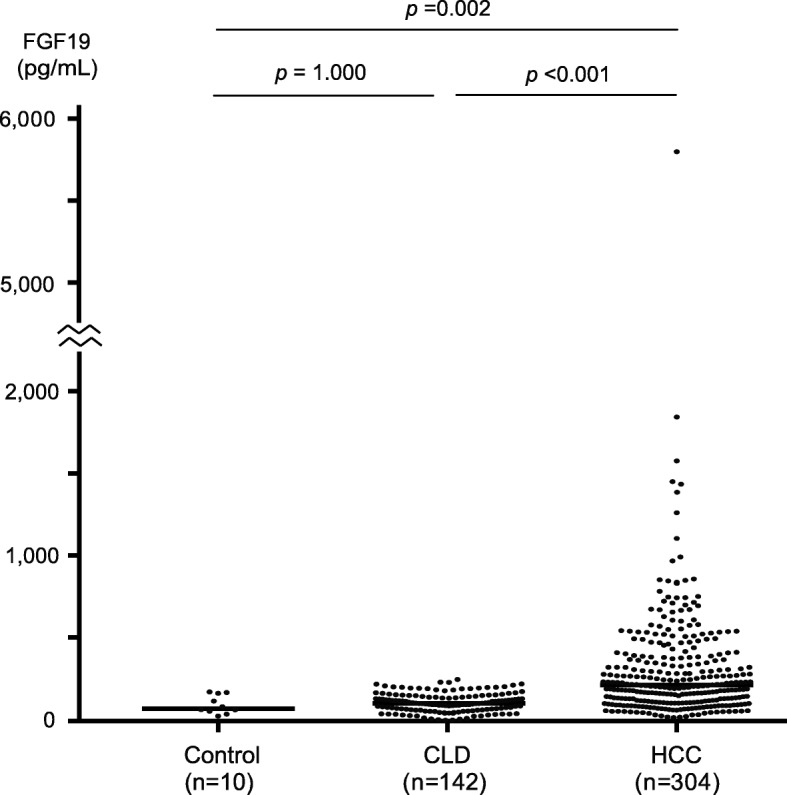
Dot plots for the serum FGF19 levels in controls, CLD patients, and HCC patients

The result of ELISA demonstrated that the median serum FGF19 levels in controls, CLD patients, and primary HCC patients were 78.8 pg/mL, 100.1 pg/mL, and 214.5 pg/mL, respectively (Fig. 1). The FGF19 levels of HCC patients ranged from 20.0 pg/mL to 5605.8 pg/mL. HCC patients showed significantly higher serum FGF19 levels than controls (*p* = 0.002) and CLD patients (*p* < 0.001). Although CLD patients showed a tendency to have higher serum FGF19 levels than controls, the difference was not statistically significant.

### ROC analysis

**Fig. 2:**
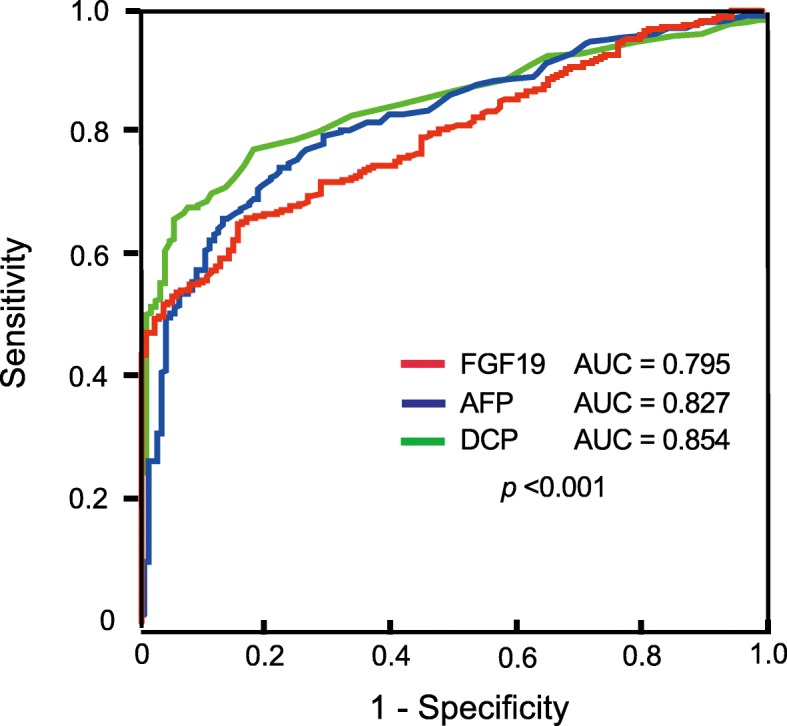
ROC curves of FGF19**,** AFP, and DCP for HCC diagnosis

To compare the diagnostic ability of FGF19 and the existing markers, namely AFP and DCP, we subsequently conducted ROC analysis. The AUC values for FGF19, AFP, and DCP for HCC detection were 0.795, 0.827, and 0.854, respectively (Fig. 2).

### Diagnostic value of FGF19 compared to those of AFP and DCP

**Fig. 3:**
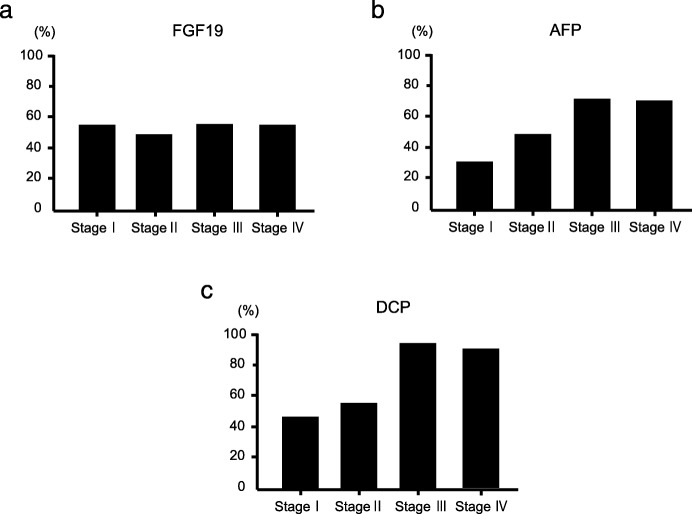
Sensitivities of FGF19, AFP, and DCP for HCC detection as per the UICC stages. Although FGF19 (**a**) remained constant at approximately 50% independent of UICC stages, AFP (**b**), and DCP (**c**) showed high sensitivity in the advanced stages but not in the early stages

We calculated the cut-off value of FGF19 as 200 pg/mL using Youden index. According to the previous studies, those of AFP and DCP were decided as 20 ng/mL and 40 mAU/mL, respectively [26, 27]. The sensitivity, specificity, positive predictive value (PPV), and negative predictive value (NPV) of FGF19 for HCC diagnosis were 53.2, 95.1, 95.9, and 48.7%, respectively (Table 2). These data appeared comparable to those of other existing markers. In particular, the sensitivity of FGF19 was significantly higher than those of other markers (55.0% for FGF19, 30.4% for AFP, and 33.3% for DCP) in patients with small HCC, defined as a solitary tumor with a diameter < 20 mm (Table 3). Thereafter, we examined the positive rate of each marker in HCC patients as per the UICC stage progression (Fig. 3). Although the sensitivities of the existing markers, including AFP, and DCP in HCC with UICC stage I were 29.1 and 44.7%, respectively, they increased with stage progression. The sensitivities of AFP and DCP in HCC with UICC stage IV were 68.2 and 90.2%, respectively. In contrast, the sensitivity of FGF19 was constant at approximately 50%, irrespective of the UICC stage (54.6% in stage I, 48.6% in stage II, 55.1% in stage III, and 54.5% in stage IV).

**Table 2:** Sensitivity, specificity, PPV, NPV, and accuracy of serum FGF19 levels and the existing markers alone and in combination in all the HCC cases

	Sensitivity (%)	Specificity (%)	PPV (%)	NPV (%)	Accuracy (%)
Single marker					
AFP	44.4	96.5	96.4	44.8	61.0
DCP	62.2	95.5	96.9	52.9	70.9
FGF19	53.2	95.1	95.9	48.7	66.6
Double markers					
AFP and DCP	73.7	91.7	95.3	61.0	77.6
AFP and FGF19	76.0	91.5	95.1	64.0	80.9
DCP and FGF19	81.3	91.0	95.4	68.8	82.5
Multiple markers					
AFP, DCP, and FGF19	87.5	87.2	94.0	76.3	85.7

**Table 3:** Sensitivity, specificity, PPV, NPV, and accuracy of serum FGF19 levels and the existing markers alone and in combination in small HCC cases

	Sensitivity (%)	Specificity (%)	PPV (%)	NPV (%)	Accuracy (%)
Single marker					
AFP	30.4	96.5	80.7	74.1	74.9
DCP	33.3	95.5	79.3	73.4	71.1
FGF19	55.0	95.1	84.4	79.9	82.0
Double markers					
AFP and DCP	53.6	91.7	77.1	79.2	75.4
AFP and FGF19	68.1	91.5	79.7	85.5	83.9
DCP and FGF19	66.7	91.0	79.3	84.0	79.1
Multiple markers					
AFP, DCP, and FGF19	75.4	87.2	75.3	87.2	80.1

### Supplementary effect of FGF19 on HCC detection when used with the existing markers

**Fig. 4:**
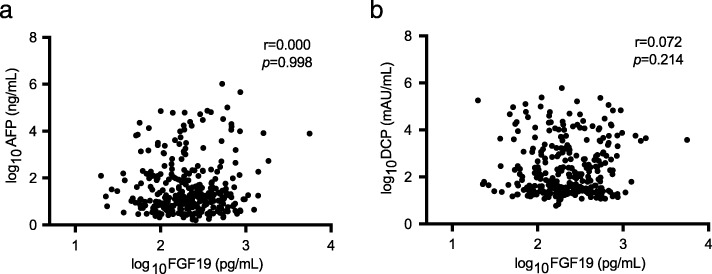
Correlation of the serum FGF19 levels with AFP and DCP. Spearman’s rank correlation coefficient analyses showed no correlation of serum FGF19 levels with AFP (**a**) or DCP (**b**)

We estimated the relationship between FGF19 and the existing markers. Spearman’s rank correlation coefficient analysis demonstrated no significant correlation of the serum FGF19 level with AFP or DCP (Fig. 4). Therefore, we examined an additional effect of FGF19 measurement on the existing markers of HCC detection. As expected, the additional FGF19 measurement resulted in increased sensitivity and NPV and a mild decrease in the specificity and PPV. In all the HCC cases analyzed, the addition of FGF19 measurement to AFP or DCP increased the sensitivity from 44.4 to 76.0% and 62.2 to 81.3%, respectively. Although the sensitivity of the combined use of AFP and DCP was 73.7%, the added measurement of FGF19 increased the sensitivity to 87.5% (Table 2). A similar marked trend was observed in patients with small HCC (Table 3). In the analyses of small HCC, the addition of FGF19 to AFP or DCP increased the sensitivity from 30.4 to 68.1% and 33.3 to 66.7%, respectively. It is noteworthy that the sensitivity of the combined use of AFP and DCP was only 53.6%, while that with AFP, DCP, and FGF19 was up to 75.4%. Thus, not only FGF19 measurement, but also the combined analyses of FGF19 and the existing markers could contribute to HCC diagnosis, especially in patients with small HCC.

### FGF19 as a marker for response to treatment and recurrence prediction

**Fig. 5:**
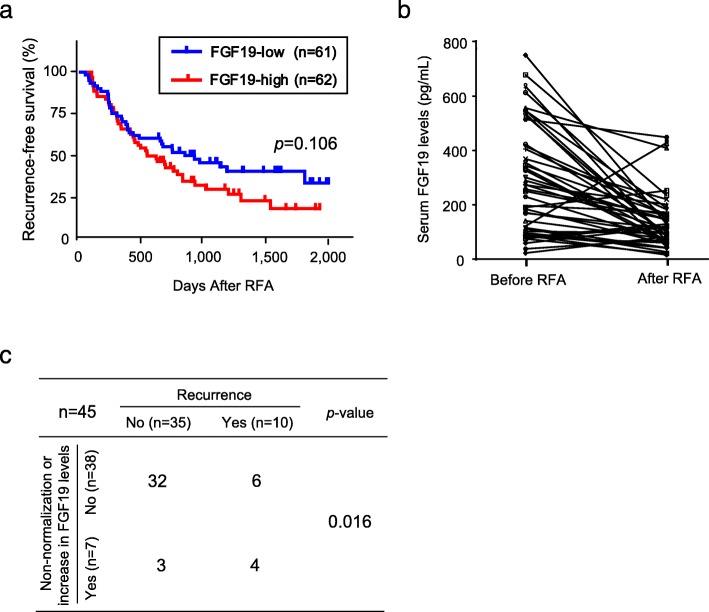
Utilities of FGF19 as a biomarker for recurrence prediction. (**a**) Cumulative RFS rate based on serum FGF19 levels before ablation therapy. (**b**) Changes in the serum FGF19 levels after the ablation therapy against HCC. (**c**) Relationship between non-normalization and an unexpected increase in the FGF19 levels after the ablation therapy and early recurrence

Among the 304 HCC patients, 123 were treated with RFA, those had achieved complete response based on post-RFA imaging findings. Subsequently, they were subjected to the prognostic analyses. Although there were no statistical differences, Kaplan-Meier analyses showed a trend that RFS in patients with high serum FGF19 levels (≥200 pg/mL) is shorter than those in patients with low serum FGF19 levels (*p* = 0.106, Fig. 5a). We then focused on the changes in FGF19 levels. For 45 of the 123 patients, paired serum samples were prepared for the further analyses (Fig. 5b). Among them, 25 patients with high serum FGF19 levels (≥200 pg/mL) showed a decrease in the FGF19 levels diminished after the ablation therapy. Although 5 patients maintained elevated FGF19 levels after the ablation, 2 patients developed recurrence within a year of RFA. Among the 20 HCC patients with low FGF19 levels, 2 showed an increase in the serum FGF19 levels (≥200 pg/mL) after the treatment. Subsequently, 1 patient experienced recurrence within a year of RFA. The recurrence rates in patients with non-normalization and an unexpected increase in the FGF19 levels after the ablation therapy were significantly higher than those in patients without them (*p* = 0.016, Fig. 5c).

## Discussion

Recent advances in research that has used next-generation sequencers has enabled the detection of genomic aberrations [28]. Thus, substantial chromosomal and genetic abnormalities of the driver genes have been reported in a variety of cancers, including HCC [29]. Among them, focal amplification of the *FGF19* gene, located on chromosome locus 11q13, has been detected in 20% of all clinical HCC samples [30]. In contrast, immunohistochemical analyses have demonstrated that FGF19 overexpression is observed in approximately 50% of all HCC cases [31]. These findings indicates that FGF19 overexpression in HCC tissues may not be accompanied by its copy number gain. Considering that FGF19 is a serum secretory protein produced by HCC cells in an autocrine loop fashion, we investigated the efficacy of serum FGF19 as a tumor marker.

First, the serum FGF19 levels of HCC patients were measured using a sandwich ELISA. As expected, the serum FGF19 levels in HCC patients were significantly higher than those in controls (*p* = 0.002) and in CLD patients (*p* < 0.001). Given that FGFs are associated with pulmonary fibrosis and renal fibrosis [32, 33], we investigated the influence of the FGF19 serum levels on liver fibrosis. Consequently, Spearman’s rank correlation coefficient analyses showed no relationship of FGF19 with hyaluronic acid or FIB-4 index (data not shown). These results indicate the possibility that FGF19 functions as a marker of HCC rather than that of severe fibrosis.

Ideal biomarkers should be highly sensitive and specific to enable early detection of HCC. Our results demonstrated that the sensitivity of FGF19 was highest among that of the existing markers, followed by that of DCP in all HCC patients, and was highest in patients with small liver cancer. Existing markers, such as AFP and DCP, often remain in the normal range in HCC patients, not only in the early stage, but also in the advanced stage [34, 35]. Although the sensitivities of AFP and DCP were 29.1 and 44.7% in HCC with UICC stage I, respectively, there was an increasing trend with stage progression. In contrast, the sensitivity of FGF19 was constant at approximately 50%, irrespective of the UICC stage. It is noteworthy that in patients with stage I, the sensitivity of FGF19 was significantly higher than those of AFP and DCP.

The specificity of FGF19 was comparable to those of the other markers in both all the patients with HCC and patients with small HCC. However, the FGF19 levels in CLD patients were mildly but not significantly elevated as compared to those in controls. Although bile acid is produced from cholesterol in the liver, hepatic bile acid synthetic levels and its secretion are tightly regulated by enterohepatic circulation [36]. However, cholestasis and liver dysfunction increase the concentrated bile acid in both the blood and bile and induce hepatocyte injury [37]. Similar to HCC cells, normal hepatocytes produce FGF19 in an autocrine fashion to protect hepatocytes from the cytotoxicity of bile acid in mice model [38]. Considering that FGF19 inhibits bile acid synthesis via the downregulation of cholesterol 7 alpha-hydroxylase (Cyp7a) [39], a mild increase in the serum FGF19 levels of CLD patients may be responsible for the negative feedback of elevated serum bile acid levels.

Because there was no significant correlation of the serum FGF19 levels and the existing markers such as AFP or DCP, we then examined whether FGF19 measurement in addition to that of these markers could improve the HCC detection rate. As expected, the addition of FGF19 measurement increased the sensitivity of HCC detection. Of importance, 79 of the 304 cases (26.0%) showed negativity for AFP and DCP, 43 cases (14.1%) could be detected using FGF19 measurement. Moreover, from the 32 cases (out of 69 cases, 46.4%) negative for AFP and DCP in patients with small HCC, 15 cases (21.7%) showed abnormal elevation in the serum FGF19 levels. Whereas AFP and DCP are markers that are comparatively highly specific for HCC, increased serum FGF19 levels have been reported in several types of cancers [40]. This is the point which requires attention.

Unlike the existing markers, FGF19 is a functional protein that is responsible for essential intracellular signal of HCC. FGF19 plays an important role in the proliferation of both, tumor cells and endothelial cells; therefore, it is believed to be promising therapeutic target molecules [41]. In fact, anti-FGF19 antibody treatment is reported to reduce the growth of colon tumor xenografts and prevent HCC development in FGF19 transgenic mice [42]. Furthermore, molecular-targeted drugs for advanced HCC, including sorafenib, regorafenib, and lenvatinib, are categorized as multikinase inhibitors; FGFR4, a receptor for FGF19, is one of the most important therapeutic targets [43, 44]. The activation in FGF19/FGFR4 signaling contributes to sorafenib resistance; therefore, abnormal FGF19 production may be associated with the treatment effect of these drugs [45]. Further analyses are necessary to clarify this issue.

## Conclusion

FGF19 functions as a tumor marker for HCC detection, especially for small HCC. The combined use of FGF19 with AFP and DCP increases the diagnostic accuracy of HCC. Further, FGF19 could be a marker for monitoring the therapeutic effect and making a prognostic prediction.

## Data Availability

Data is available upon request.

## Abbreviations

AFP: Alpha-fetoprotein
AFP-L3: *Lens culinaris* agglutinin-reactive fraction of alpha-fetoprotein
ALT: Alanine aminotransferase
AUC: Area under the curve
CLD: Chronic liver disease
CLEIA: Chemiluminescence enzyme immunoassay
DCP: Des-gamma-carboxy prothrombin
FGF19: Fibroblast growth factor 19
FGFR4: Fibroblast growth factor receptor 4
HBV: Hepatitis B virus
HCC: Hepatocellular carcinoma
HCV: Hepatitis C virus
IQR: Interquartile range
NPV: Negative predictive value
OS: Overall survival
PPV: Positive predictive value
RFA: Radiofrequency ablation
RFS: Recurrence-free survival
ROC: Receiver operating curve
UICC: Union for international cancer control
